# Menstrual Pattern following Tubal Ligation: A Historical Cohort Study

**DOI:** 10.22074/ijfs.2015.4605

**Published:** 2015-12-23

**Authors:** Shahideh Jahanian Sadatmahalleh, Saeideh Ziaei, Anoshirvan Kazemnejad, Eesa Mohamadi

**Affiliations:** 1 Department of Midwifery and Reproductive Health, Faculty of Medical Sciences, Tarbiat Modares University, Tehran, Iran; 2 Department of Biostatistics, Faculty of Medical Sciences, Tarbiat Modares University, Tehran, Iran; 3 Department of Nursing, Faculty of Medical Sciences, Tarbiat Modares University, Tehran, Iran

**Keywords:** Historical Cohort Study, Tubal Ligation, Menstrual Disorders, Pictorial Blood Loss Assessment Chart

## Abstract

**Background:**

Tubal ligation (TL) is recommended for women who have completed their family planning. The existence of the menstrual disorders following this procedure has been the subject of debate for decades. This study was conducted to identify the relationship between tubal ligation and menstrual disorders.

**Materials and Methods:**

A historical cohort study was carried out on 140 women undergoing tubal ligation (TL group) and on 140 women using condom as the main contraceptive method (Non-TL group). They aged between 20 and 40 years and were selected from a health care center in Rudsar, Guilan Province, Iran, during 2013-2014. The two groups were comparable in demographic characteristics, obstetrical features and menstrual bleeding pattern using a routine questionnaire. A validated pictorial blood loss assessment chart (PBLAC) was also used to measure the menstrual blood loss.

**Results:**

Women with TL had more menstrual irregularity than those without TL (24.3 vs. 10%, P=0.002). Women with TL had more polymenorrhea (9.3 vs. 1.4%, P=0.006), hypermenorrhea (12.1 vs. 2.1%, P=0.002), menorrhagia (62.9 vs. 22.1%, P<0.0001) and menometrorrhagia (15.7 vs. 3.6%, P=0.001) than those without TL. There is a significant difference in the PBLAC score between women with and without TL (P<0.0001). According to logistic regression, age odds ratio [(OR=1.08, con- fidence interval (CI):1.07-1.17, P=0.03)], TL (OR=5.95, CI:3.45-10.26, P<0.0001) and cesarean section (OR=2.72, CI:1.49-4.97, P=0.001) were significantly associated with menorrhagia.

**Conclusion:**

We found significant differences in menstrual disorders between women with and without TL. Therefore, women should be informed by the health providers regarding the advantages and disadvantages of TL before the procedures.

## Introduction

Some women who have completing family planning choose tubal ligation (TL) as a method of contraception (1). Menstrual disorder is one of the problematic effects of TL, although the results of related studies have been inconsistent and inconclusive (2, 3).

The occurrence of abnormal bleeding after TL was first described by Williams et al. (4). It has been hypothesized that ligation may increase incidence of menstrual disturbances among women receiving TL. Several studies about the side-effects of TL on menstrual function have been conducted (5, 6), yet the existence of a post TL syndrome has been debated. The term post tubal ligation syndrome (PTLS) was first reported in the early 1950s based on the results of a study in which the effect of menstrual disorders on some of somatic and psychological symptoms were evaluated (4). Although based on the conjecture, it has been hypothesized that TL may result in low blood flow to the ovaries, leading to impairment of follicular growth and altered gonadotropin signal and ovarian hormone levels, resulting in menstrual disorders (7). Abnormalities reports associated with TL surgery include the entire spectrum of menstrual disorders, such as: more frequent menstrual periods, irregular menstrual cycles, menorrhagia, metrorrhagia, spotting, dysmenorrhea and oligomenorrhea (8). However, some studies (2, 9) showed no increase in menstrual disorders in women undergoing TL as compared with a control group.

Resolving the debate about menstrual disorders after TL is important for safeguarding women’s health. Therefore, we compared the occurrence of menstrual disorders in women with and without TL. This is a pioneer study in Iran investigating type of menstrual disorders in women with TL.

## Materials and Methods

For this historical cohort study, first a pilot study was conducted on 60 women. Then, using the appropriate formula with α at 0.05 and 1-β at 0.95, it was found that a sample size of 130 women was needed for each group. Therefore, 140 women undergoing TL at least a year ago, and 140 women using condom as contraceptive method at least for 3 months were assigned as TL and non-TL groups, respectively. All participants were recruited from a healthcare center in Rudsar, Guilan Province, Iran, between 2013 and 2014.

The inclusion criteria were as follows: i. Multiparous, ii. 20-40 years of age, iii. Free of chronic diseases, including diabetes, hypertension, thyroid and cardiovascular diseases, iv. Free of any gynecological diseases and v. At least three normal cycles before TL.

We compared the distribution of demographic characteristics, obstetrical features and menstrual bleeding pattern between two groups using a routine self-administered questionnaire. A validated pictorial blood loss assessment chart (PBLAC) was also used for the evaluation of menstrual blood loss (MBL) (10). This chart records the amount of daily menstrual bleeding by noting the number of clots, the amount of staining on each pad or tampon. Everyone completed their charts for one menstrual cycle. All patients used the same sanitary products.

In order to build a prediction model and to find the most important factors affecting menorrhagia, we used backward logistic regression analysis in which a p value of 0.15 was used as an entry criterion, whereas a p value of 0.10 was the threshold for a variable to stay in the model.

The outcome variable was menorrhagia. The following variables were included in the logistic regression model: age, age at menarche, parity, body mass index (BMI), education status, TL status (women with or without TL) and method of delivery.

This study was approved by the Ethics Committee of the Tarbiat Modares University. All women participated voluntarily and provided a signed informed consent.

### Definitions and Terminology for Menstrual Pattern

Normal menstrual: A menstrual interval of 21-35 days and a flow duration of 7 days or less are considered normal (11).
Menstrual cycle length: The number of days from the beginning of one menstrual period to the beginning of the next one is defined as menstrual cycle length (11).
Menstrual irregularities: A menstrual interval shorter than 21 days and longer than 35 days is defined as menstrual irregularities. Amount of bleeding is varied (5).
Oligomenorrhea: Bleeding intervals longer than 35 days is defined as oligomenorrhea (12).
Polymenorrhea: A menstrual interval shorter than 21 days is defined as polymenorrhea (13).
Hypermenorrhea: Flow more than 7 days is considered as hypermenorrhea (11).
Metrorrhagia: Metrorrhagia is defined as vaginal bleeding occurring between the expected menstrual periods (3).
Menorrhagia: Menorrhagia is defined as a PBLAC score of ≥100 (14). Length of menstruation cycle is not important in diagnosis of menorrhagia because this definition is not valid by itself (15).
Menometrorrhagia: Excessive and prolonged bleeding occurring irregularly is defined as menometrorrhagia (11).

#### Statistical analysis

All statistical analyses were performed by the Statistical Package for the Social Sciences (SPSS) version 20.0 (SPSS Inc., USA). Student’s t test and chi-square test were carried out to reveal the statistical differences between the groups. We used logistic regression to determine the risk factors associated with menorrhagia. Odds ratio (OR) and 95% confidence interval was also calculated for each factor. A P value less than 0.05 was considered to be statistically significant.

### Results

By considering the inclusion criteria, 140 tubal ligated and 140 non-tubal ligated subjects were evaluated for menstrual disorders. Table 1 gives the characteristics of TL and non-TL groups. There are no significant differences in the age, age of menarche, BMI, parity, education status and the method of delivery between women with TL compared to non-TL group. However, there is a significant difference in PBLAC score for menstrual loss between the two groups. The mean score of PBLAC is statistically significant in women with TL compared to non-TL group (137.72 ± 90.91 vs. 87.91 ± 51.06, P<0.0001, Table 2). Table 2 displays findings regarding the participants’ menstruation disorders. Women with TL had more menstrual irregularity than those without TL (24.3 vs. 10%, P=0.002). Women with TL had more polymenorrhea (9.3 vs. 1.4%, P=0.006), hypermenorrhea (12.1 vs. 2.1%, P=0.002), menorrhagia (62.9 vs. 22.1%, P<0.0001) and menometrorrhagia (15.7 vs. 3.6%, P=0.001) than those without TL.

The mean duration of TL was 4.6 ± 1.4 years. The duration of TL had no effect on menorrhagia. The mean duration of TL is not statistically significant in the women with menorrhagia as compared to the nonmenorrhagia (4.57 ± 1.50 vs. 4.80 ± 1.45, P=0.37) (The data are not shown).

In the logistic regression model, age (OR=1.08, CI:1.07-1.17, P=0.03), TL (OR=5.95, CI: 3.45- 10.26, P<0.0001) and cesarean section (OR=2.72, CI:1.49-4.97, P=0.001) are positively associated with menorrhagia (Table 3).

**Table 1:** Comparison of demographic and personal characteristics between TL and non-TL groups


Parameters	Non-TL	TL	Sig

Women’s age (Y)	35.45 ± 4.51	36.22 ± 3.14	0.09^a^
Partner’s age (Y)	38.92 ± 4.41	38.15 ± 3.10	0.59^a^
Age of menarche (Y)	12.65 ± 1.34	12.73 ± 1.38	0.71^a^
Parity	2.21 ± 0.46	2.32 ± 0.53	0.36^a^
BMI (Kg/m^2^)	27.67 ± 4.53	28.37 ± 5.16	0.21^a^
Educational level			
Under diploma	70 (50)	74 (52.9)	0.14b
Diploma and high school diploma	70 (50)	66 (47.1)	
Method of delivery			
Normal vaginal delivery	50 (35.7)	40 (28.6)	0.22^b^
Caesarian section	90 (64.3)	100 (71.4)	
Previous contraceptive method used			
Pill	3 (2.1)	5 (3.6)	
Condom	127 (90.7)	117 (83.6)	0.22^b^
Other*	10 (7.1)	18 (12.9)	


TL; Tubal ligation, ^a^; T test, ^b^; Chi-square test, BMI; Body mass index and ^*^; This category included withdrawal and natural family planning or the rhythm method.

**Table 2:** Comparison of menstrual disorders between groups


Parameters	Non-TL	TL	Sig

Menstrual irregularities*	14 (10)	34 (24.3)	0.002^a^
Oligomenorrhea^*^	12 (8.6)	21 (15)	0.12^a^
Polymenorrhea^*^	2 (1.4)	13 (9.3)	0.006^a^
Hypermenorrhea^*^	3 (2.1)	17 (12.1)	0. 002^a^
Metrorrhagia^*^	9 (6.4)	12 (8.6)	0.64^a^
Menorrhagia^*^	31 (22.1)	88 (62.9)	<0.0001^a^
Menometrorrhagia^*^	5 (3.6)	22 (15.7)	0.001^a^
PBLAC score^**^	87.91 ± 51.06	137.72 ± 90.91	<0.0001^b^


^*^; n (%), ^**^; Values are mean ± SD, ^a^; Chi-square test, ^b^; T test, TL; Tubal ligation, and PBLAC; Pictorial blood loss assessment chart.

**Table 3:** Logistic regression analysis of 280 women for menorrhagia


Variables	OR (95% CI)*	Sig

Age	1.08 (1.07-1.17)	0.03
TL status		
Yes	5.95 (3.45-10.26)	<0.0001
No	1**	
Method of delivery		
Cesarean section	2.72 (1.49-4.97)	0.001
Normal vaginal delivery	1**	
Constant	0.007	0.001


*; OR, CI (OR; Odds ratio, CI; Confidence interval), **; Reference category and TL; Tubal ligation.

## Discussion

Numerous investigators have evaluated the impact of TL on menstrual cycle characteristics. Although the literature on the effects of TL and menstrual disorders are comprehensive, they have been inconsistent (2, 6, 16, 17).

Our results indicated that sterilized women were more likely to experience an increase in polymenorrhea, hypermenorrhea, menorrhagia, and menometrorrhagia and to have an irregular menstrual cycle when compared with the other group.

Some studies showed a significant increase in incidence of menstrual disorder in women undergoing TL when compared with a control group (4, 16, 17). Increased duration (hypermenorrhea) and amount of bleeding (menorrhagia) have been reported by Shain et al. (18). TL has been considered as the cause of menstrual abnormalities by damaging the ovary (19), including acute increase in pressure in the utero-ovarianarterial loop (20).

Peterson et al. (5) found women undergoing TL experienced a shortened interval between menses and a decrease in volume of menstrual flow and in bleeding days as compared with related values in non-sterilized women. However, Shobeiri and Atashkhoii (9) concluded that TL does not cause menstrual disorders. Several other studies concluded that the duration of bleeding, volume of menstrual flow, menstrual cycle length and cycle irregularity are similar in women with and without tubal legation (2, 6). Although it has been hypothesized that menstrual disorders are caused by the damaging effect of TL on ovarian function through an increase in pressure within the utero-ovarian arterial circulation or disruption of the ovarian blood supply, some researchers have not observed an alteration in ovarian function (5, 6). In addition, laboratory studies comparing women before and after TL have found no constant abnormalities in ovarian function (5), indicating no difference in luteinizing hormone (LH), follicle stimulating hormone (FSH) and estradiol (E_2_) levels in women undergoing TL when compared with a non-TL group (6).

Menorrhagia is identified as the most common bleeding disorders (21). Several methods were used to measure menstrual blood loss, like alkalin hematin method that is a cheap, acceptable, easy and relatively accurate test (22); however, we preferred to measure indirectly the blood loss using the PBLAC (10). We found a significant increase in PBLAC score for menstrual blood loss in women undergoing TL when compared with a non-TL group. Several studies showed that there was no significantly difference regarding menorrhagia between the case and control groups (9, 23). In another study by Wilcox et al. (17), they reported heavy menstrual flow (41%) after 5 years following TL.

We evaluated patient characteristics, age, TL and cesarean section as predictors of menorrhagia. Our findings showed that age, TL and cesarean section are positively associated with menorrhagia.

Some studies also indicated that age could be considered as a risk marker for menorrhagia and irregular menstrual bleeding (10, 24). The most significant changes in late reproductive age include a decrease in anti-Mullerian hormone (AMH) and in early cycle inhibin B levels. A decline in inhibin B results in an increase in FSH levels (25). Burger et al. (26) showed that increasing FSH levels are associated with normal or higher E_2_ concentrations. Ultimately, these changes cause menstrual disorders. The mechanisms leading to menstrual disorders may involve the temporary ovarian nonresponsiveness to FSH stimulation and the critical numbers of follicles. No ovarian response may occur for several days with increasing FSH levels, but finally a follicle starts to develop, leading to a hyper-respond and higher concentration of E_2_ (25).

The present study also assessed the relationship between method of delivery and menorrhagia. Our results indicated menorrhagia was more common in women with history of caesarian section. Harlow et al. (6) concluded that menstrual irregularity, length of menstruation, length of cycle and flow volume are similar in women with and without TL, but women with a history of cesarean section and TL experienced an increase in volume of menstrual flow compared with women who did not undergo TL. Uppal et al. (24) reported similar findings. Regnard et al. (27), however, found no relationship between the method of delivery and menstrual disorders. Osser et al. (28) have also referred to endometrial defects at cesarean scar site and the weakness of uterine contractions as a cause for menstrual disorders.

The present study shows that menstrual disorders were more common in women with TL. There are still many important questions to be investigated about probable effects of TL on menstrual disorders. This study conveys an important message that TL may influence irregular menstruation and menorrhagia. Hence, women should be informed and instructed by health providers such as midwifes and gynecologists regarding the advantages and disadvantages of TL. Definitely, this database is not large enough to give precise conclusion and needs further supports for long-term follow-up for menorrhagia in patient undergoing TL.

Our findings suggest that menorrhagia and menstrual irregularities are more prevalent than previous reports about Iranian women with TL. This is a pioneer study in Iran investigating type of menstrual disorders in women with TL. The different studies have showed that the relationship between TL and menstrual disorders is a complex process influenced by multiple factors. Therefore, biological, physiological, psychological, cultural, behavior, ethnicity, climate, and religious conditions as well as lack of knowledge of women about TL may affect the present findings.

Most of women participating in this study had no information about other types of sterilization and their side effects. Consequently, we were unable to evaluate the effect of particular method of TL on menstrual disorder, indicating limitation in our study. On the other hand, as our study was a historical cohort, no documents were available about surgical skills used for TL, which shows another limitation in this study.

## Conclusion

Overall, this study showed that TL is a cause of menstrual disorders. However, we need more evidence based on cohort studies to confirm the results of the present study.
